# Integration of machine learning and meta-analysis identifies the transcriptomic bio-signature of mastitis disease in cattle

**DOI:** 10.1371/journal.pone.0191227

**Published:** 2018-02-22

**Authors:** Somayeh Sharifi, Abbas Pakdel, Mansour Ebrahimi, James M. Reecy, Samaneh Fazeli Farsani, Esmaeil Ebrahimie

**Affiliations:** 1 Department of Animal Science, College of Agriculture, Isfahan University of Technology, Isfahan, Iran; 2 Department of Animal Science, Iowa State University, Ames, Iowa, United States of America; 3 School of Basic Sciences, University of Qom, Qom, Iran; 4 Department of Chemical Engineering, Ferdowsi University of Mashhad, Mashhad, Iran; 5 School of Medicine, The University of Adelaide, Adelaide, Australia; 6 Institute of Biotechnology, Shiraz University, Shiraz, Iran; 7 Division of Information Technology, Engineering and the Environment, School of Information Technology and Mathematical Sciences, University of South Australia, Adelaide, South Australia, Australia; 8 School of Biological Sciences, Faculty of Science and Engineering, Flinders University, Adelaide, South Australia, Australia; University of Illinois, UNITED STATES

## Abstract

Gram-negative bacteria such as *Escherichia coli* (*E*. *coli*) are assumed to be among the main agents that cause severe mastitis disease with clinical signs in dairy cattle. Rapid detection of this disease is so important in order to prevent transmission to other cows and helps to reduce inappropriate use of antibiotics. With the rapid progress in high-throughput technologies, and accumulation of various kinds of ‘-omics’ data in public repositories, there is an opportunity to retrieve, integrate, and reanalyze these resources to improve the diagnosis and treatment of different diseases and to provide mechanistic insights into host resistance in an efficient way. Meta-analysis is a relatively inexpensive option with good potential to increase the statistical power and generalizability of single-study analysis. In the current meta-analysis research, six microarray-based studies that investigate the transcriptome profile of mammary gland tissue after induced mastitis by *E*. *coli* infection were used. This meta-analysis not only reinforced the findings in individual studies, but also several novel terms including responses to hypoxia, response to drug, anti-apoptosis and positive regulation of transcription from RNA polymerase II promoter enriched by up-regulated genes. Finally, in order to identify the small sets of genes that are sufficiently informative in *E*. *coli* mastitis, the differentially expressed gene introduced by meta-analysis were prioritized by using ten different attribute weighting algorithms. Twelve meta-genes were detected by the majority of attribute weighting algorithms (with weight above 0.7) as most informative genes including *CXCL8* (*IL8*), *NFKBIZ*, *HP*, *ZC3H12A*, *PDE4B*, *CASP4*, *CXCL2*, *CCL20*, *GRO1(CXCL1)*, *CFB*, *S100A9*, *and S100A8*. Interestingly, the results have been demonstrated that all of these genes are the key genes in the immune response, inflammation or mastitis. The Decision tree models efficiently discovered the best combination of the meta-genes as bio-signature and confirmed that some of the top-ranked genes -*ZC3H12A*, *CXCL2*, *GRO*, *CFB-* as biomarkers for *E*. *coli* mastitis (with the accuracy 83% in average). This research properly indicated that by combination of two novel data mining tools, meta-analysis and machine learning, increased power to detect most informative genes that can help to improve the diagnosis and treatment strategies for *E*. *coli* associated with mastitis in cattle.

## Introduction

Bovine mastitis is an inflammatory disease with clinical and subclinical forms which result in significant economic losses due to negative impacts on animal welfare [1–3], productive [4–6] and reproductive performances [7, 8], poor milk quality [9], increased workload [10], early culling [1, 11], and high treatment costs [12]. Clinical mastitis was detected in almost 25% of the 9.3 million dairy cows present in the USA every year; a quarter of them were removed/sold from the herd, and approximately less than 5% of all cows died as a result of mastitis [13]. Environmental pathogens including coliforms are the major contributors to clinical mastitis causing acute inflammation with clinical signs in dairy cows, which however may be self-healing by eventually eradicating the invader [14], are occasionally fatal [15]. Nevertheless, self-care is often associated with a longer duration of infection, lower milk yield, and the potential for pathological changes in the mammary gland [16].

There is evidence that mastitis-causing pathogens use various mechanisms to induce cell pathways. Hence, the identification of pathogens is of major importance in order to correct actions, prevent transmission to other cows, reduce the risk of appearance of chronic infections, and helps to reduce inappropriate use of antibiotics, antimicrobial resistance and cost of treatment [17–19]. Disease-causing genes [20] and biomarkers help to improve diagnosis, prognosis, and monitoring of responses to therapy [21]. Genes coding for proteins such as Haptoglobin (HP), Serum Amyloid A (SAA) [22], Cathelicidin antimicrobial peptide (CAMP) [23], and Lingual antimicrobial peptide (LAP) [24] have been identified as potential biomarkers for mastitis detection. The performance of the most mastitis detection systems do not satisfy the high accuracy required for practical clinical mastitis detection systems [25, 26]. Potential to include several biomarkers on one test strip to enhance the diagnostic efficiency is an aim of developmental research. Antibiotic therapy should be chosen based on mastitis pathogen and the type of mastitis [27, 28]; therefore, biomarker discovery with the focus on specific pathogens will be useful. The efficacy of antibiotic and/or anti-inflammatory treatment in mastitis is still a topic of scientific debate, and studies on treatment value in clinical cases show conflicting results [29, 30]. Moreover, efforts to find other therapy methods such as homeopathic treatment had no success in this disease [31]. Identification of disease-causing genes that underlie complex traits such as susceptibility to mastitis is the goal of many genetic and biomedical studies, which provides mechanistic insights into host resistance in addition to improving the diagnosis and treatment of the disease. The amplitude of the inflammatory response is mainly dependent on individual cow factors, and different animals will respond inconsistently to *Escherichia coli* (*E*. *coli)* infection [32, 33]. Combining the results of independent studies with a related hypothesis using meta-analysis, as a relatively inexpensive option with good potential to increase the statistical power and the generalizability of single-study analysis, can bypass the challenges associated with individual variations, and strengthen the mildest data perturbations [34, 35]. In the previous meta-analysis studies, different aspects of mastitis disease have been investigated. Genini *et al*. (2011) identified a common transcriptional response to different pathogens in the mammary glands of several species [36]. Younis *et al*. (2016) investigated differences in transcriptional response between *E*. *coli* and *Staphylococcus aureus* strains infections and also between lipopolysaccharide (LPS), and *E*. *coli*-induced mastitis [37].

In the current study, for the first time, two novel data mining tools, meta-analysis and machine learning, were integrated to detect differentially expressed gene (DE)s and prioritize them to identify the most informative genes in response to *E*. *coli* mastitis. Attribute weighting algorithm (AW)s and Decision tree model (DT)s are the most widely used approaches in machine learning. Various algorithms of AW or feature selection give weight to features and allow the variable set to be reduced in size, thereby creating a more manageable set of attributes for modeling and attribute ranking [38, 39]. Decision tree models predict the value of a discrete dependent variable within a finite set of independent variables [40]. We used various DTs to classify samples in datasets for confirmation of AWs. The high efficiency and applicability of several well-known AWs and DTs have been demonstrated previously [41–44].

## Material and methods

The following steps were performed in this article: 1. Identifying the suitable microarray studies of bovine mammary gland infected with *E*. *coli*, extracting the data from studies, preparing, normalizing, and annotating the individual studies; 2. Analyzing individual studies and then combining the studies-specific *p-values* with rOP meta-analysis method; 3. Fulfilling the functional enrichment analysis on the DEs introduced by meta-analysis; 4. Applying 10 different AWs on standardized expression values of meta-genes in all samples to rank and select the most important genes and making 10 new datasets based on the selection of attributes; and 5. Utilizing various DTs to classify samples in datasets for confirmation of AWs

### Microarray datasets

PubMed central ("https://www.ncbi.nlm.nih.gov/pubmed/"Accessed January 2016) and Google Scholar ("https://scholar.google.com/"Accessed January 2016) were searched by using “*Bos Taurus* [organism]”,“Mastitis” and “*Escherichia coli*” keywords. Microarray gene expression data were retrieved from either, GEO of NCBI ("https://www.ncbi.nlm.nih.gov/gds/" Accessed January 2016) or ArrayExpress of EMBL_EBI ("https://www.ebi.ac.uk/arrayexpress/"Accessed January 2016). Twelve studies matched these search criteria. Upon additional review, only six studies were selected for further analysis as they all used the Affymetrix bovine GeneChipTM ("http://www.affymetrix.com/index.affx" Accessed February 2016). Information of these studies are shown in Table 1. Studies were excluded from the meta-analysis for the following reasons: had non-commercial platforms, which incompletely overlap the Affymetrix arrays, therefore would significantly reduce the number of genes after matching and/or they had incomplete annotation or no valid citation. Affymetrix Bovine Genome Array platform contains 24,128 probe sets to measure global transcript abundance (Bovine.na.36, March 2016). From these probe sets, 19,192 ones, which had an associated gene symbol, were used in the analysis reported here. The Bovine Genome Array annotation is available from NetAffx Analysis Centre ("http://www.affymetrix.com/support/technical/annotationfilesmain.affx Accessed December 2016"). Only samples infected by *E*. *coli* without any treatment and appropriate controls were used in this analysis. The study by Brand *et al*. which was mentioned in Table 1, had samples from animals with either high or low susceptibility to mastitis [45]. Only data from the highly susceptible animals were used in this analysis. As sampling times after infection differed among experiments, each sampling time was considered as a separate study. A total of 130 mammary gland samples (57 healthy and 73 infected) of 15 retrieved datasets from 6 studies were included in the differential expression analysis (Table 1).

**Table 1:** Summary of the microarray datasets employed in meta-analysis in this study.

Accession number	Citation	Treatment time^a^ (h)	Pathogen	Challenge/ Inoculum dose	Kind of experiment	Preparation of bacteria	Samples (ctr:tr)^b^
GSE15025	[46]	6	*E*. *coli* 1303	500 CFU^c^	in vivo	Live	5:5
		24					5:5
GSE24217	[47]	24	*E*. *coli* K2BH2	20–40 CFU	in vivo	Live	9:12
		192					14:14
GSE24560	[45]	1	*E*. *coli* 1303	100 μL solution	in vitro	heat-inactivated	3:5
		6					3:5
		24					4:4
GSE25413	[48]	1	*E*. *coli* 1303	10^7^ particles/ml	in vitro	heat-inactivated	3:3
		3					3:3
		6					3:3
		24					3:3
GSE32186	[49]	6	*E*. *coli* 1303	10^7^ particle/ml	in vitro	heat-inactivated	3:3
		6^d^					3:3
GSE50685	[29]	24	*E*. *coli* ECC-Z	100 CFU	in vivo	Live	2:2
		48					3:3

^a^ Time of sampling after infection

^b^ Number of healthy samples: number of treatment samples

^c^Colony Forming Unit

^d^Cells were harvested either 30h (short waiting experiment) or 60 h (long waiting experiment) after the start of the trial.

### Pre-processing of microarray datasets

The quality of each dataset was explored by PCA analysis and box plots before and after normalization, as previously described [50–52]. Quartile normalization and summarization were performed on individual datasets by log scale Robust Multi-array Average (RMA) [53] as implemented in R Affy package [54].The Affymetrix Bovine GeneChipTM has multiple probes (or probe sets) that represent the same genes. Therefore, gene matching was necessary for these probe sets/genes. Among all possible probe IDs for a given gene, the probe ID with the largest Inter-Quartile Range (IQR) of expression value was selected to represent that gene. In order to reduce the false discovery rate of microarray data analysis, we removed approximately 10% of the non-expressed genes based on the small average expression values across the majority of studies, and approximately 10% of the non-informative genes that had minimal amounts of variation. Final dataset (Fd) was used for the next meta-analysis process. The MetaDE package in R (version 1.0.5) was used for matching and filtering procedures [55].

### Meta-analysis

Here, we utilized transcriptome data from 6 independent studies that were different in employed techniques (in vivo versus in vitro), methods of bacterial preparation (live *E*. *coli* versus heat-inactivated *E*. *coli*), strains of *E*. *coli* (1303, K2BH2 and ECC-Z) and also different doses of Challenge (see Table 1). Differences in the response to bacterial challenge of the mammary epithelial cells in vivo and in vitro have been characterized previously [56, 57]. It has been shown that virulence factors of heat-inactivated pathogens are different from those of active pathogens [45]. It has been illustrated that phenotypic properties of strains from different phylogroups are likely to be different [58]. For this reasons, we used meta-analysis based on *p-values* because this method permits us to join related studies with heterogeneous data [59]. For each meta-analysis, it is possible to apply different purposes with different approaches. In the current study, we considered investigating genes, which commonly up/down expressed in all studies related to *E*. *coli* mastitis.

**Fig 1:**
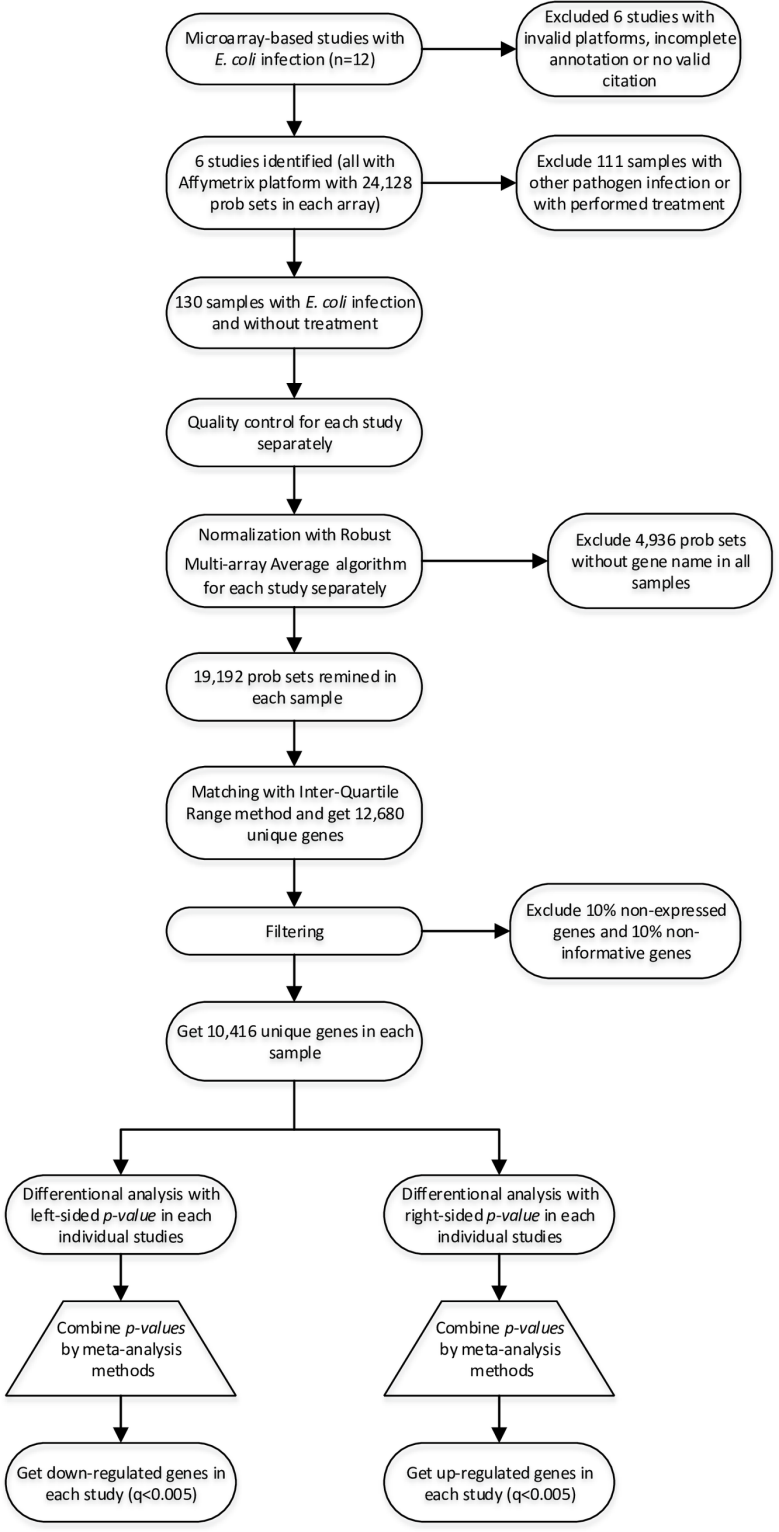
All processes including selection of studies, pre-processing of datasets (quality control, normalization, summarization and preparation of datasets), individual differential analysis and performing a meta-analysis to achieve differentially expressed genes (meta-genes).

Here, at first, expression levels of mastitis and healthy samples for each gene were compared by using a moderated Student’s t-test implemented to run on Fd by MetaDe package [55]. We used a one-tailed *p-value* analysis in each study to specify the direction of the alternative hypothesis to identify up- and down-regulated genes after meta-analysis. The *p-values* of each dataset were used in the r^th^ ordered *p-value* (rOP) meta-analysis method. We used r^th^ = 5 to combine *p-values* in order to detect DEs in 5 smallest *p-values* among all datasets (out of 15 datasets) [60]. A separated meta-analysis performed on right-sided *p-values*, and left-sided *p-values* offer up- and down-regulated genes, respectively. A false discovery rate adjustment for multiple testing with cut off value of 0.005 (one tailed) was performed as described by Benjamini and Hochberg [61]. All individual data analyses and meta-analyses were performed in R program (version 3.3.1) using the MetaDE package (version 1.0.5). Differentially expressed gene(s) identified by meta-analysis (meta-gene(s)) were used for machine-learning process. A flow diagram has been prepared to better understanding of all processes in an attempt to achieve meta-genes (see Fig 1).

### Functional enrichment analysis

The meta-genes were submitted to functional annotation tool of Dataset for Annotation, Visualization and Integrated Discovery program, version 6.8, (DAVID, http://david.abcc.ncifcrf.gov/home.jsp) in order to identify the biological processes, cellular components and molecular functions [62, 63].

We analyzed the gene ontologies for up- and down-regulated meta-genes separately. The gene ontology (GO) terms generated by modified Fisher Exact test and terms with *p-values* better than 0.05 were selected.

### Attribute weighting algorithms

After meta-analysis, 885 genes showed DEs between healthy and induced mastitis samples, based on Benjamini & Hochberg adjustment *p-value* correction (q<0.01). To improve the ability to detect the most informative genes, we used a two-step standardization procedure proposed by Yoon *et al*. (2006) on meta-genes including within-array standardization (array-specific Z-score calculation) followed by the gene-specific multi-array standardization (gene-specific Z-score calculation) [64]. Subject feature (categorized as healthy and mastitis) was set as the target or label variable and standard expression value of meta-genes was set as feature or attribute, which were classified as continuous data. This new dataset (Metad), was used to import into RapidMiner Studio software (RapidMiner 7.0.001 Gmbh). A supplemental spreadsheet file shows this dataset (see S1 Table).

Ten different AWs consisting of PCA, Uncertainty, Relief, Chi Squared, Gini Index, Deviation, Rule, Gain Ratio, Information Gain, and SVM [65] were applied on the list of meta-genes. We ranked meta-genes based on the number of AT algorithms which indicate that gene (attribute) is important (weight above 0.7) with respect to the subject (mastitis and healthy). Ten new datasets produced by trimming the Metad based on a weight above 0.7 given by each AW (Attribute Selection), as well as the Metad (11 datasets in total), used as input for DT models.

### Decision tree models

Sixteen Tree Induction models including: Decision Tree, Random Tree, Tree Stump, Tree and Random Forest models, each model with 4 different criteria Accuracy, Gain Ratio, Gini Index and Information Gain were applied on eleven datasets including the original Metad and 10 datasets generated by the10 AWs as described previously [65]. The Decision tree model s was applied to find patterns between important genes. The models were run with a minimal size of two for all leaves, a minimal gain of 0.1 to produce a split, and a maximal tree depth of 20. A confidence level of 0.25 was selected for the pessimistic error calculation for pruning[42]. The performance of different models in prediction of the target variable (healthy and mastitis) based on attribute variables (standardized expression of meta-genes) was used to calculate model efficiency. Accuracy was calculated by taking the percentage of correct predictions over the total number of samples (130 samples). A ten-fold cross-validation algorithm with stratified sampling was used to build the trees. Furthermore, an average of ten runs were used to calculate the performance percentage [66].

The PRISMA checklist is included as S2 Table.

## Results

### Meta-analysis increase power to detect DEs

From the 19,192 probe sets on the Affymetrix Bovine Genome Array, which contained annotation, 12,860 unique genes were identified after matching. Following the filtering step, the meta-analysis was applied on 10,416 probes. 885 meta-genes were differentially expressed, of which 143 genes were down-regulated and 742 genes were up-regulated (one-tailed, q<0.005). We prepared a supplemental spreadsheet file that contains more additional information (see S3 Table). In these meta-genes, 291 genes never showed a significant q-value in any of the individual studies, most likely due to the relatively small sample sizes of those individual studies (see S4 Table). The results provide a strong evidence that meta-analysis has improved the ability of DEs detection.

### Functional annotation clustering analysis of meta-genes revealed several novel themes

In order to understand the functional significance of the identified meta-genes, GO enrichment analysis was performed using the DAVID program. We had more focus on biological process pathways. The *p-values*<0.05 were used to determine statistically significant categories. Up-regulated genes mainly enriched the biological processes terms were associated with the immune response, defense responses, inflammation, chemotaxis, acute phase protein, protein degradation and proteolysis, growth and death of cell, response to wounding and cell signaling pathways. Product of up-regulated genes was mostly localized in plasma membrane and extracellular region based on cellular component analysis.

Down-regulated genes mainly enriched terms related to fatty acid metabolism and lipid biosynthesis including cholesterol, sterol, terpenoid biosynthesis and metabolic process. All components of GO terms related to up- and down-regulated genes were shown in supplemental spreadsheet files (see S5 and S6 Tables respectively).

### Attribute weighting algorithms were used to rank meta-genes

Various AWs were employed to identify the important genes. In the AWs, normalized data were used to run the models. It was expected that all weights would be between 0 and 1.0 value, closer to 1 is an indication that a given gene is an important attribute. *CXCL2* gene (Chemokine (C-X-C motif) ligand 2) was the most important gene pointed out by 70% of the AWs (7 from 10 AWs); followed by *CXCL8*, *CFB*, *ZC3H12A*, *CCL20*, *NFKBIZ*, *S100A9*, *S100A8*, *PDE4B*, *CASP4* and *HP*. A table containing the meta-genes with all weights given by 10 AWs was shown in a supplemental spreadsheet file (see S7 Table). A complete list of high relevant genes that were confirmed by the majority of AWs (with a weight above 0.7) is presented in Table 2. In order to run DTs, 10 new datasets based on attribute selection with weights above 0.7 in each AWs were also generated.

**Table 2:** The most important attributes (differentially expressed genes introduced by meta-analysis) ranked based on 10 attribute weighting algorithms (AWs), including PCA, Uncertainty, Relief, Chi-Squared, Gini Index, Deviation, Rule, Gain Ratio, Information Gain, and SVM.

Attribute (Gene symbol)	Gene name (alias)	The number of AWs that indicate the attribute is important (weight above 0.7)
*CXCL2*	chemokine (C-X-C motif) ligand 2 (GRO3)	7
*CXCL8*	C-X-C motif chemokine ligand 8 (IL-8, IL8)	6
*GRO1*	chemokine (C-X-C motif) ligand 1 (CXCL1, MGSA)	6
*CFB*	complement factor B (BF)	6
*ZC3H12A*	zinc finger CCCH-type containing 12A	6
*CCL20*	C-C motif chemokine ligand 20	5
*NFKBIZ*	NFKB inhibitor zeta (MAIL)	5
*S100A9*	S100 calcium binding protein A9	5
*S100A8*	S100 calcium binding protein A8	5
*PDE4B*	phosphodiesterase 4B	5
*CASP4*	caspase 4, apoptosis-related cysteine peptidase (CASP13)	5
*HP*	haptoglobin	5

Decision tree models identified gene bio-signatures that can discriminate mastitis from healthy samples. Sixteen different DTs were applied to eleven datasets. The minimum and maximum performances were 53.08% and 86.5%, respectively (Table 3).

**Table 3:** Comparison of performance percentage of 16 Decision tree induction models run on 11 datasets (10 datasets generated by trimming the Metad based on a weight above 0.7 given by each AWs plus the Metad) of differentially expressed genes introduced by meta-analysis response to mastitis disease.

Models Datasets	Random Forest Accuracy (%)	Random Forest Gini index (%)	Random Forest Information Gain (%)	Random Forest Gain Ratio (%)	Random Tree Accuracy (%)	Random Tree Gini index (%)	Random Tree Information Gain (%)	Random Tree Gain Ratio (%)	Tree Stump Accuracy (%)	Tree Stump Gini index (%)	Tree Stump Information Gain (%)		Tree Stump Gain Ratio (%)	Decision Tree Accuracy (%)		Decision Tree Gini index (%)	Decision Tree Information Gain (%)	Decision Tree Gain Ratio (%)
Chi Squared	78.46	83.08	86.15	84.62	70.00	70.77	76.92	74.62	73.85	83.08		79.23	79.23	83.08	83.85		81.54	83.08
Deviation	66.92	63.08	58.46	58.46	60.00	64.62	62.31	53.08	60.77	62.31		56.92	56.92	64.62	62.31		58.46	55.38
Gini Index	83.85	82.31	78.46	82.31	70.77	76.15	80.00	73.85	73.85	83.08		79.23	79.23	86.92	84.62		83.08	82.31
Information Gain	82.31	84.62	82.31	83.08	72.31	76.15	75.38	74.62	73.85	83.08		79.23	79.23	86.92	86.15		83.08	81.54
Gain Ratio	83.85	84.62	84.62	82.31	73.85	70.77	75.38	70.00	79.23	83.08		79.23	79.23	84.62	79.23		78.46	80.77
PCA	77.69	78.46	78.46	73.85	58.46	66.15	63.08	66.92	70.00	73.08		72.31	72.31	71.54	80.00		83.08	79.23
Relief	80.77	80.77	82.31	81.54	70.77	71.54	83.85	75.38	82.31	83.08		82.31	82.31	83.08	80.00		75.38	81.54
Rule	75.38	79.23	75.38	80.00	63.85	64.62	66.92	66.92	68.46	80.00		64.62	64.62	76.92	76.92		73.85	81.54
SVM	83.08	83.85	78.46	83.85	74.62	72.31	73.85	66.92	82.31	83.08		82.31	82.31	79.23	76.92		80.00	80.00
Uncertainty	83.85	85.38	84.62	80.77	72.31	76.15	77.69	73.08	73.85	83.08		79.23	79.23	83.08	78.46		80.77	79.23
Metad	78.46	79.23	78.46	75.38	76.92	67.69	67.69	67.69	73.85	83.08		79.23	79.23	79.23	80.00		76.92	76.92

**Fig 2:**
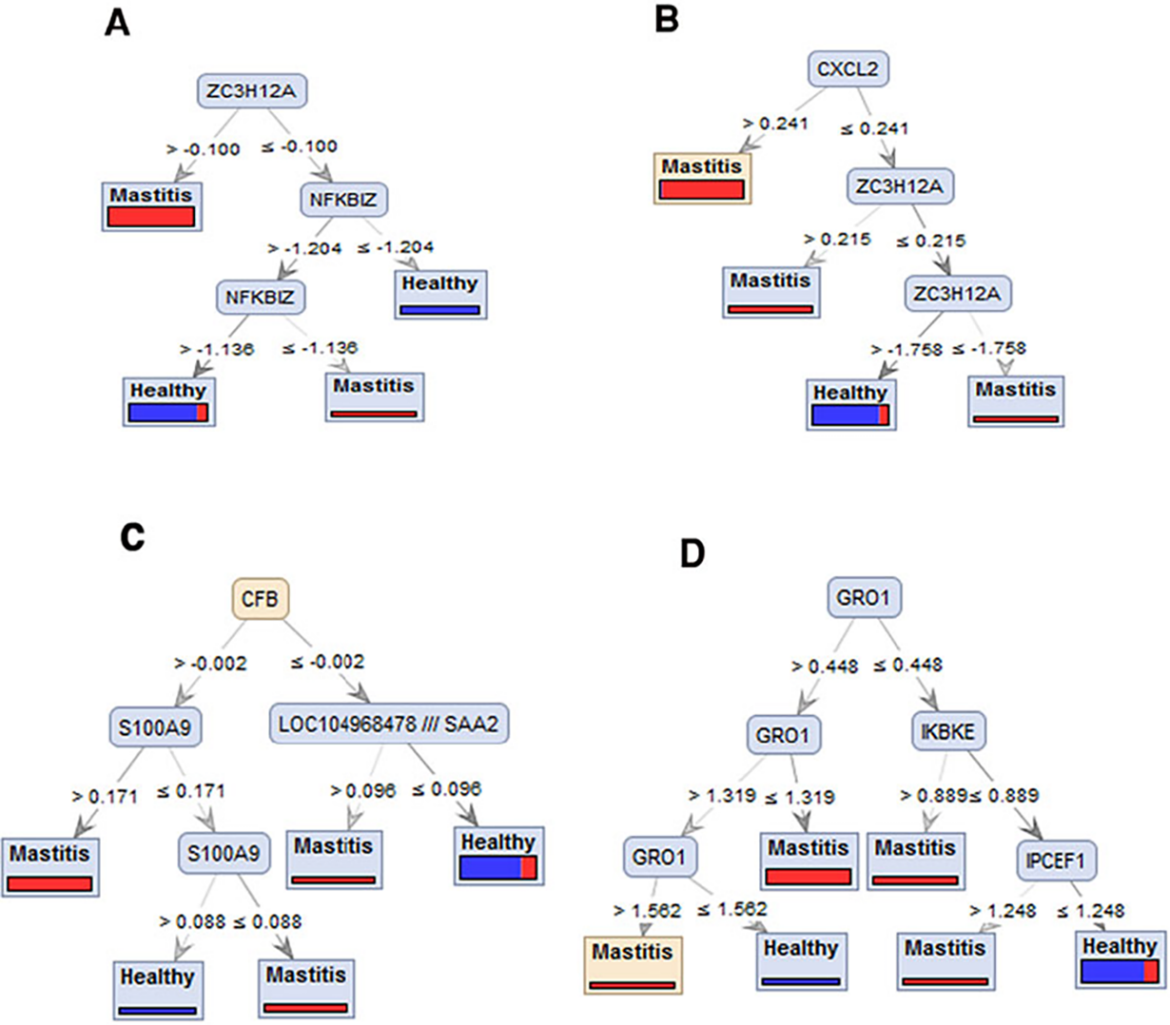
The architecture of different Decision tree models in predicting mastitis and healthy samples, based on the differentially expressed genes introduced by meta-analysis (A) Random Forest model with Gini Index criterion run on SVM dataset. (B) Random Forest model with accuracy criterion run on Gini Index dataset (C) Random Forest with Information Gain criterion run on Relief dataset and (D) Random Forest model with Gain Ratio criterion run on SVM dataset.

The architecture of selected threes generated by DTs has shown in Fig 2. This selection was based on the size of tree, display the role of top-ranked genes in the classification of samples and performance percentages of trees in prediction of label of samples as healthy or mastitis based on standard expression value of meta-genes. We generated these trees by performing of Random Forest models with Gini Index, Accuracy, Information Gain and Gain Ratio criterion run on SVM (A), Gini Index(B) Relief (C) and SVM (D) datasets respectively. As shown in Fig 2 (A), *ZC3H12A* gene has potential biomarker performance. When the value of *ZC3H12A* gene was greater than -0.100, the cases fell into the mastitis class. Moreover, when the value was equal to or lower than -0.100, and the value of *NFKBIZ* gene was lower than -1.204, a sample fell into the healthy class. In contrast, when the value of last feature was equal or higher than -1.138, the sample fell into the healthy class. Otherwise, a sample fell into the mastitis class with an accuracy of 83.85%, indicating that from the 130 samples, 110.5 were correctly categorized between mastitis and healthy class. In Fig 2, in the same way, *CXCL2* in B part, *CFB* in C part and *GRO1* in D part were at the peak of trees and have potential biomarker performance with 83.85%, 82.31%, and 83.85% accuracy respectively.

## Discussion

With the rapid progress in high-throughput technologies and accumulation of various kinds of ‘-omics’ data in public repositories, there is an opportunity to retrieve, integrate, and re-analyze them to identify the most important genes and biomarker candidates in an efficient way [67–70]. Based on definition of biomarker, a “good” biomarker as an indicator must be specific for a disease and should remain unchanged by unrelated disorders. Moreover, reliable and reproducible biomarker quantifications must be demonstrated [17].

Here, we performed a meta-analysis on series of microarray gene expression datasets in order to enhance the power of analysis to identify genes that may be significantly involved in response to *E*. *coli* mastitis in dairy cows. Meta-analysis confirmed the most important findings in individual studies such as induction of the pathways related to immune response, inflammation, cytokines and chemokines signaling, acute phase proteins, proteolysis, response to wounding, apoptosis and cell signaling. It also suppressed several aspects of basic epithelial biology including extracellular matrix biosynthesis, mammary gland development markers and epidermis morphogenesis such as cholesterol, sterol and terpenoid biosynthesis [29, 45–49]. Importantly, based on our results, *E*. *coli* infection causes down-regulation of genes encoding lipid biosynthesis enzymes including *ALOX15*, *FASN*, *GPAM*, *TM7SF2* that are involved in milk production [37]. Generally, in infection, host metabolism is suppressed because the tissue has to divert energy to fight infection. Moreover, up-regulated meta-genes enriched novel biological pathways including responses to hypoxia, positive regulation of transcription from RNA polymerase II promoter and anti-apoptosis agents.

Low oxygen (O_2_) environments are created by pathophysiological conditions including sites of infection and inflammation. In addition, pyruvate accumulation caused by inhibition of lipid metabolism has indeed been shown to stimulate hypoxia signaling in mastitis disease in dairy cattle [71]. In the previous studies, the results have demonstrated that stress-response genes such as those responsible to immune-response pathways were enriched in paused RNA polymerase II [72]. For this reason, and due to this point that RNA polymerase II is essential for the transcription of many genes which up-regulated genes during *E*. *coli* infection, induced expression of genes related to positive regulation of transcription from RNA polymerase II promoter is necessary. Macrophages are the key players in innate immunity, and because of their crucial role in immunity, regulation of monocyte/macrophage lifespan is important in both physiological and pathological processes. Anti-apoptotic genes such as *Bcl2* family has been shown to be involved in the survival of monocytes/macrophages through enhancing the resistance of macrophages against various apoptotic stimuli [73].

In the current research, for the first time, the machine-learning approach were used to prioritize meta-genes to find the most important genes in response to *E*. *coli-*induced mastitis. The top-ranked meta-genes- *CXCL8 (IL8)*, *NFKBIZ*, *HP*, *CXCL2*, *CCL20*, *GRO1*, *ZC3H12A*, *PDE4B*, *CASP4*, *CFB*, *SA00A9*, *SA00A8-* that were listed in Table 2 play an important role in the immune defense, inflammation, and/or chemotaxis. Inflammatory chemokine interleukin-8 (IL-8), one of the most widely studied chemokines, is a critical inflammatory mediator and plays an important role in neutrophil migration into bovine mammary glands during mastitis [74, 75]. Furthermore, previous studies demonstrated IL-8 as an antibody therapeutic target in inflammatory diseases in human [76] and bovine mastitis [74].

IκBζ (also known as Molecule possessing ankyrin-repeats induced by lipopolysaccharide (MAIL) and INAP), encoded by the *NFKBIZ* gene, is a member of the nuclear IκB family of proteins that act as transcriptional regulators via association with nuclear factor kappa B (NF-κB)) [77]. The critical role of IκBζ signaling in the regulation of immune responses has been revealed previously [78, 79]. Like other IκB proteins, IκBζ has inhibitory effects on the transcription of inflammatory genes regulated by NF-κB such as tumor necrosis factor (TNF)-α, interleukin-1 (IL-1) [77, 80] and IL-17A production from CD4+ T cells [81]. Furthermore, it has been demonstrated that IκBζ is indispensable for the expression of a subset of genes activated in TLR/IL-1R signaling pathways [77]. Toll-like receptors (TLRs) recognize various bacterial cell wall components such as LPS, peptidoglycan (PGN) and lipopeptides, and trigger the inflammatory and immune responses against pathogens [82]. Investigations have revealed that function and gene polymorphisms of *NFKBIZ* can be introduced as potential markers of mastitis resistance in dairy heifers [83].

Already abbreviated Haptoglobin (HP), an acute phase protein mostly secreted by the liver, is synthesized within the mammary gland through stimulation by pro-inflammatory stimuli as it is in the liver [84]. HP has been introduced as a sensitive inflammatory marker for acute mastitis by numerous studies [84–86].

Pro-inflammatory cytokines, chemokines such as CXCL2, CCL20, and GRO1(CXCL1) have important roles in immune responses due to modulation of leukocyte infiltration (neutrophils and monocytes). CXCL2 has been determined as a biomarker of the inflammatory reaction previously [87]. It has been suggested that CXCL1 can be used as therapeutic targets, therapeutics, or biomarkers in mastitis [88]. According to our result and validation with DTs, as shown in Fig 2, *CXCL2* and *GRO1* have good abilities to separate mastitis and healthy samples with 83.85% accuracy; and they are good candidates to distinguish *E*. *coli* mastitis as a biomarker.

Zinc finger protein, ZC3H12A, has been shown as TLR-inducible gene to modulate LPS-induced inflammatory response [89]. It is also an RNase essential for the control of immune responses by regulating mRNA decay [90]. As shown in Fig 2 and based on DTs, *ZC3H12A* also has been identified as a potential biomarker for *E*. *coli* mastitis with 83.85% accuracy. However it needs more investigations at the protein level to be considered as a biomarker.

The PDE4B2 is the short isoform of PDE4 isoenzyme family. PDE4 is cAMP-specific and the dominant *PDE* in inflammatory cells. Inhibition of PDE4 elevates intracellular cAMP levels, which inhibit the activity of promoters such as NF-κB and down-regulation of the inflammatory responses by reducing the expression of TNF-α and other pro-inflammatory cytokines, while increasing anti-inflammatory cytokines such as IL-10 [91]. Interestingly, PDE4 inhibition is used as therapeutics for the treatment of inflammatory diseases in numerous studies [92, 93]

Caspases are a family of cysteine proteases that are highly conserved in multicellular organisms, functioning as central regulators of apoptosis [73]. Caspase-4 is classified as inflammatory caspases [94]. CASP4 has been shown to bind with LPS with high specificity and affinity directly and it is an innate immune receptor for intracellular LPS [94, 95]. It has been demonstrated that caspase-4 plays an important role in the classical LPS induced TLR4-signaling pathway, leading to *NF-κB* dependent transcriptional up-regulation and secretion of important cytokines and chemokines in innate immune signaling in human monocytic cell [94]. Remarkably, CASP4 represents a new candidate for pattern recognition in immunity [95].

Complement factor B (CFB) an acute phase plasma protein is central to the action of the innate immune system in response to inflammation and infection and plays a role in B-cell activation and the cytotoxic reaction [86, 96, 97]. Research in bovine has demonstrated that the complete complement system can be found in colostrum, and components of the system are also present in the milk [97]. At present, attention is being focused on using acute phase proteins such as haptoglobin, serum amyloid A. [85, 98, 99], as biomarkers for the diagnosis of mastitis. However they are non-specific markers of the inflammatory process. *CFB* has been confirmed by DTs with 82.31% accuracy (Fig 2) and it may be a good candidate for the diagnosis of *E*. *coli* mastitis.

The role of last two top-ranked genes, *SA00A9* and *SA00A8*, are inducing chemotaxis and adhesion of neutrophils [100] and play an important role in the innate immunity and tissue repair [101]. Moreover, these genes were identified as biomarkers for acute inflammation in infused and autoimmune disease [102, 103].

Due to the fact that the performances of the most mastitis detection systems do not satisfy the high accuracy required for practical clinical mastitis detection [25, 26], potential to include several biomarkers on one test strip or commercial kit might enhance the diagnostic efficiency of mastitis. Therefore, antibiotic therapy can, therefore, be chosen based on the mastitis pathogen and the type of mastitis. These results are valuable bioinformatics findings that need more laboratory based-studies to confirm.

## Conclusions

This finding showed that the meta-analysis based on a large amount of original data represents an important contribution to our understanding of most informative genes for *E*. *coli* mastitis in cattle. Furthermore, this research properly indicated that the combination of machine learning with meta-analysis provides an opportunity to obtain a better resolution of the most important genes that might provide a more robust bio-signature and thereby may be good biomarker candidates. Our results provide the basis for strategies to improve the diagnosis and treatment of the *E*. *coli* mastitis in the dairy cow.

## Supporting information

S1 Table Standard expression value of differentially expressed genes achieved by meta-analysis (Meta-genes) were set as features or attributes, this new dataset (Metad) was used to import into RapidMiner Studio software. (XLSX)

S2 Table Table is the PRISMA Checklist. (DOC)

S3 Table Differentially expressed genes identified after meta-analysis (one-tailed q<0.005). (XLSX)

S4 Table Investigation of count of individual studies which show significant *q-value* (Benjamini & Hochberg corrected p-value, one tailed q<0.005) for differentially expressed genes achieved after meta-analysis (meta-genes). (XLSX)

S5 Table The gene ontology (GO) information based on modified Fisher Exact test analysis (*p-value*<0.05), revealed on up-regulated genes achieved by meta-analysis (meta-genes). (XLSX)

S6 Table The gene ontology (GO) information based on modified Fisher Exact test analysis (*p-value*<0.05), revealed on down-regulated genes achieved by meta-analysis (meta-genes). (XLSX)

S7 Table All weights give after applying 10 attribute weighting algorithms (AW)s on differentially expressed genes achieved by meta-analysis (meta-genes) and count of algorithms with given weight above 0.7. (XLSX)
