# Supplementary material for: Integration of machine learning and meta-analysis identifies the transcriptomic bio-signature of mastitis disease in cattle
Source: PLoS One. 2018 Feb 22;13(2):e0191227. doi: 10.1371/journal.pone.0191227 (PMC5823400; doi:10.1371/journal.pone.0191227)
Supplement: S2 Table — (DOC) [file pone.0191227.s002.DOC]

| **Section/topic** | **#** | **Checklist item** | **Reported on page #** |
| --- | --- | --- | --- |
| **TITLE** | | |  |
| Title | 1 | Metaanalysis of Transcriptional Responses to Mastitis-Causing Bacteria. | 1 |
| **ABSTRACT** | | |  |
| Structured summary | 2 | **Note: This is NOT a metaanalysis of clinical or preclinical trials**.  Some items in this list will not be applicable. This is a metaanalysis of data in microarray transcriptional profiling studies as available in public repositories. | 6,7 |
| **INTRODUCTION** | | |  |
| Rationale | 3 | Metaanalysis with increase the statistical power and the generalizability of single-study analysis, can bypass the challenges associated with individual variations, and robust to the mildest data perturbations. | 4,5 |
| Objectives | 4 | PICOS Participants: Individual cows and bovine mammary epithelial cell cultures. Interventions: None. Comparisons: Bacteria-infected vs. controls. Outcomes: Not applicable. Study design: mRNA analysis using Affymetrix microarrays. | 6,7,8 |
| **METHODS** | | |  |
| Protocol and registration | 5 | No review protocol | N/A |
| Eligibility criteria | 6 | Not applicable | N/A |
| Information sources | 7 | NCBI GEO datasets | 6 |
| Search | 8 | Keywords: Bos Taurus AND E. coli AND mastitis | 6 |
| Study selection | 9 | E.coli-infected vs. controls without each treatment with sampling from mammary gland tissue. Studies had non-commercial platforms, which incompletely overlap the Affymetrix arrays, incomplete annotation or no valid citation were excluded from the meta-analysis | 6 |
| Data collection process | 10 | Downloading CEL files from GEO and annotation from NetAffx. | 6 |
| Data items | 11 | Not applicable | N/A |
| Risk of bias in individual studies | 12 | Not applicable | N/A |
| Summary measures | 13 | Not applicable | N/A |
| Synthesis of results | 14 | Not applicable | N/A |

Page 1 of 2

| **Section/topic** | **#** | **Checklist item** | **Reported on page #** |
| --- | --- | --- | --- |
| Risk of bias across studies | 15 | Not applicable | N/A |
| Additional analyses | 16 | We prioritized the meta-genes using machine learning approaches | N/A |
| **RESULTS** | | |  |
| Study selection | 17 | 12 studies screened, assessed for eligibility, and 6 of them included in the analysis. | 6 |
| Study characteristics | 18 | All microarray data with E.coli-infected vs. controls without each treatment and sampling from mammary gland tissue were used. | 6 |
| Risk of bias within studies | 19 | Not applicable | N/A |
| Results of individual studies | 20 | Not applicable | N/A |
| Synthesis of results | 21 | Not applicable | N/A |
| Risk of bias across studies | 22 | Not applicable | N/A |
| Additional analysis | 23 | None | N/A |
| **DISCUSSION** | | |  |
| Summary of evidence | 24 | Meta-genes enriched the complete collection of biological processes involved in the immune defense, inflammation, and chemotaxis and also revealed a novel biological process, including “positive regulation of transcription from RNA polymerase II promoter”. For first time we prioritized the meta-genes using machine learning approach and introduced 12 genes as biomarker candidate. *CXCL8* (*IL8*) and *NFKBIZ in* that list are known to be involved in the mastitis disease and *HP* has been reported previously to be a sensitive inflammatory marker for acute E.coli mastitis. Interestingly, other introduced genes - *ZC3H12A, PDE4B, CASP4, CXCL2, CCL20, GRO1, CFB, S100A9, S100A8* - had not been previously specified to have any association with mastitis although their key functions in the immune system, inflammation and chemotaxis have been discussed. | 17,18 |
| Limitations | 25 | None obvious. | N/A |
| Conclusions | 26 | The results provide the basis for strategies to improve the diagnosis and treatment of the mastitis, provide mechanistic insights into host resistance and may lead to the reduction in the use of antibiotics in agriculture. | 19 |
| **FUNDING** | | |  |
| Funding | 27 | None. | N/A |

*From:*  Moher D, Liberati A, Tetzlaff J, Altman DG, The PRISMA Group (2009). Preferred Reporting Items for Systematic Reviews and Meta-Analyses: The PRISMA Statement. PLoS Med 6(6): e1000097. doi:10.1371/journal.pmed1000097

For more information, visit: **www.prisma-statement.org**.

Page 2 of 2
